# Liver gene regulation of hemostasis-related factors is altered by experimental snake envenomation in mice

**DOI:** 10.1371/journal.pntd.0008379

**Published:** 2020-06-01

**Authors:** Ana Teresa Azevedo Sachetto, José Ricardo Jensen, Marcelo Larami Santoro

**Affiliations:** 1 Laboratory of Pathophysiology, Butantan Institute, São Paulo, São Paulo, Brazil; 2 Department of Medical Sciences, School of Medicine, University of São Paulo, São Paulo, São Paulo, Brazil; 3 Laboratory of Immunogenetics, Butantan Institute, São Paulo, São Paulo, Brazil; Fundacao Oswaldo Cruz, BRAZIL

## Abstract

Few studies have addressed gene expression of hemostasis-related factors during acute thrombo-hemorrhagic diseases. Bites by the lanced-headed viper *Bothrops jaracaca* induce rapid hemostatic disturbances in victims, leading to systemic bleedings, thrombocytopenia and consumption coagulopathy. Although circulating levels of coagulation factors recover rapidly after administration of specific antivenom therapy, it is unclear if *B*. *jararaca* venom (BjV) upregulates the mRNA synthesis of hepatic hemostasis-related factors, or if the recovery occurs under basal conditions after the neutralization of venom components by antivenom. Thus, we aimed to investigate if BjV regulates gene expression of important hemostasis-related factors synthetized by the liver. On that account, Swiss mice were injected with saline or BjV (1.6 mg/kg b.w, s.c.), and after 3, 6 and 24 h blood samples and liver fragments were collected to analyze mRNA expression by real-time qPCR. Increased gene expression of fibrinogen chains, haptoglobin and STAT3 was observed during envenomation, particularly at 3 and 6 h. At 24h, mRNA levels of *F10* were raised, while those of *Serpinc1*, *Proc* and *Adamts13* were diminished. Surprisingly, *F3* mRNA levels were steadily decreased at 3 h. Gene expression of *Thpo*, *F7*, *F5 Tfpi*, *Mug1* was unaltered. mRNA levels of *Vwf*, *P4hb*, *F8*, *F2*, *Plg*, and *Serpinf2* were minimally altered, but showed important associations with *Nfkb1* gene expression. In conclusion, snakebite envenomation upregulates hepatic mRNA synthesis particularly of fibrinogen chains, and acute-phase markers. This response explains the fast recovery of fibrinogen levels after antivenom administration to patients bitten by *B*. *jararaca* snakes.

## Author summary

*Bothrops jararaca* snakebites induce hemostatic disturbances in man and animals, but no information is available about how the liver, the main organ involved in the synthesis of hemostasis-related factors, copes with this condition. In an effort to understand how these factors recover after *B*. *jararaca* snakebites, we evaluated the hepatic gene expression of hemostatic-related factors after experimental envenomation in mice. We noticed that a marked increase in gene expression of fibrinogen chains occurred 3 and 6 h after subcutaneous administration of *B*. *jararaca* venom, explaining thereby the rapid recovery of the coagulation disturbance after administration of specific antivenom to patients. Interestingly, gene expression of other coagulation factors consumed during envenomation, e.g. factors V, VIII, and prothrombin, was scarcely affected over time, while the expression of tissue factor mRNA was promptly decreased at 3 h. Moreover, gene expression of acute-phase proteins was modified by envenomation, as well as of the transcription factor STAT3. Gene expression of NF-κB1 was not altered significantly over time, but it was associated with the regulation of mRNA levels of various hemostasis-related genes. Our results showed that *B*. *jararaca* envenomation induces a rapid change in gene expression of some coagulation factors and acute phase proteins.

## Introduction

In 1.8–2.7 million patients bitten by various venomous snakes worldwide annually, hemostatic disorders are commonly observed [1, 2]. Although regarded as a secondary cause of acquired hemostatic disorders in hemostasis textbooks, bleedings evoked by snakebite envenomation are particularly common in patients from low-income tropical countries [3], leading to severe or fatal outcomes, e.g. life-threatening hemorrhage [4, 5], intracranial hemorrhage [6, 7], and spontaneous abortion in women [8].

Snakes from *Bothrops* genus–popularly known as jararacas in Brazil–inhabit most regions in Central and South America, where they are an important public health problem [5, 9, 10]. The venom from *Bothrops jararaca* (BjV), a snake species found in the southeastern region of Brazil, contains various enzymatic and non-enzymatic proteins–e.g., phospholipases A_2_, snake venom serine proteinases (SVSP), snake venom metalloproteinases (SVMP), L-amino acid oxidases, disintegrins and C-type lectins, among others [11]. These proteins interfere with hemostasis and disrupt the structure of vessel walls [12–14], leading to bleeding manifestations (e.g. gingival bleeding, hematuria, gastrointestinal bleeding, petechiae, ecchymosis, etc) in patients envenomed by these snakes [15, 16]. Besides, BjV also induces a phlogistic reaction at the site of venom inoculation, which may deteriorate progressively, leading to local necrosis as an ultimate outcome [17]. This exacerbated inflammatory reaction is considered essential to the burst of local and systemic inflammatory mediators, and synthesis of acute-phase proteins (APP) observed in patients and mice inoculated with BjV [18, 19].

Hemostatic abnormalities evoked by *B*. *jararaca* snakebites include direct proteolytic activity of snake venom enzymes on fibrinogen, prothrombin, and factors X, VIII and V, in a mechanistic process that is distinct from the extrinsic and intrinsic coagulation pathways, and by a feedback mechanism of these activated factors, especially meizothrombin or thrombin, which ultimately leads to activation and consumption of coagulation factors in circulation, as well as exacerbated secondary fibrinolysis [12, 20–23]. Thrombocytopenia and platelet dysfunction are also observed during *B*. *jararaca* envenomation [16, 22, 24], but they are independent of coagulation activation [21, 25]. Gradual fibrinogen consumption during snakebite envenomation causes hypofibrinogenemia or afibrinogenemia, leading to a prolongation of whole blood coagulation time (WBCT)–which is a sign of snake envenomation [26]. In health centers that treat snakebites, the modified WBCT is the most frequently used laboratory test to evaluate how intensely snake venoms have impaired the blood coagulation cascade [24, 26, 27].

Due to the effectiveness of specific serotherapy to treat coagulopathy and thrombocytopenia evoked by *B*. *jararaca* envenomation [15], restoration of blood coagulability and platelet counts occurs rapidly in patients, between 6 and 18 h after antivenom administration. However, it is not known how this process occurs, whether hemostasis-related factors are regenerated under basal hepatic conditions by simple neutralization of circulating antivenom, or whether pathophysiological events that take place during envenomation upregulate gene expression. Indeed, the current international consensus [28], based on previous clinical studies [29, 30], suggests that an additional dose of antivenom should be administered six hours after the first dose if the WBCT remains prolonged. However, this indication needs further scientific evidence, as the rate of coagulation factor synthesis is unknown under conditions of envenomation. Under basal conditions, it is known that fibrinogen has a half-life of *ca*. 90 h and is synthetized at a rate of ca. 34 mg/kg/day [31]. Thus, in a completely defibrinogenated 70-kg patient, taking an average plasma volume of 50 mL/kg, plasma fibrinogen concentration would reach mean levels around 68 mg/dL after 24 hours under basal fibrinogen synthesis condition, if venom had been completely neutralized by antivenom. In fact, this value is below the inferior hemostatic level of 100 mg/dL for fibrinogen [32], not explaining thereby the rapid restoration of fibrinogen levels observed in patients bitten by *B*. *jararaca* [15]. We hypothesized that the expression of hemostasis-related genes was somehow stimulated after envenomation in the liver, the main organ involved in the synthesis of hemostatic factors, even in the absence of antivenom administration. To address this hypothesis, taking into account both the inflammatory and hemostatic events occurring during envenomation that may influence hepatic gene expression, we evaluated mRNA expression levels in livers of mice injected with BjV [33], a model that replicates the hemostatic manifestations observed in bitten patients.

## Methods

### Materials

Lyophilized venom from adult specimens of *B*. *jararaca* snakes was obtained from the Laboratory of Herpetology, Butantan Institute (Sistema Nacional de Gestão do Patrimônio Genético e do Conhecimento Tradicional Associado, SisGen AF375C2). *Bothrops* antivenom was kindly donated by Butantan Institute (lot: 1305077). All reagents were of analytical grade or better.

### Ethics statement

Male Swiss mice (n = 30), weighing 30–35 g, were obtained from the Animal Facility of Butantan Institute, and were maintained with free access to food and water. The experimental procedures involving mice were in accordance with National Guidelines, and were approved by the Institutional Animal Care and Use Committee from Butantan Institute (CEUAIB 4388061115) and from the School of Medicine, University of São Paulo (protocol 188/15). All procedures involving animals were in accordance with the National Guidelines from Conselho Nacional de Controle de Experimentação Animal (CONCEA) [34]. Blood and liver samples were collected from the same mice used previously [33].

### Envenomation

Mice were randomly assigned to two experimental groups (n = 5 animals/group/time interval). Mice from the saline control group were injected subcutaneously (s.c.) with sterile saline alone (vehicle), and mice from the BjV group were injected with freshly diluted BjV (1.6 mg/kg body weight, s.c.). The dose of BjV was based on a previous report [33], and it evoked characteristic hemostatic disturbances.

After 3, 6 or 24 h, mice were anesthetized with isoflurane (induction and maintenance at 2.5%) for the collection of blood and tissues. Blood was collected from the caudal vena cava into plastic syringes, and added to plastic flasks containing 269 mM Na_2_EDTA for complete blood cell counts. Blood was also added to plastic flasks containing the anticoagulant CTAD (75 mM trisodium citrate, 42 mM citric acid, 139 mM dextrose, 15 mM theophylline, 3.7 mM adenosine, 0.2 mM dipyridamole, and 2 μM imipramine), and plasma was obtained by centrifugation at 2500 *g* for 15 min at 4°C. *Bothrops* antivenom (1 vol. to 100 vol. of blood) was added to all flasks to halt the *in vitro* activity of BjV after blood collection. Liver fragments from the central region of the left lateral lobe (50–100 mg) were collected, immersed in 500 μL of RNAlater (Thermo, USA) and stored at -80°C until used.

### Hemostatic parameters

Platelet counts were obtained using an automated cell counter (BC-2800 Vet, Mindray, China) and plasma fibrinogen levels were measured with a colorimetric assay [35].

### RNA isolation and cDNA synthesis

Liver fragments were removed from RNALater, immersed in 1 mL of TRIzol reagent, incubated for 5 min at room temperature, and disrupted using FastPrep-24 (#6004–500, MP Biomedicals) for 60 s at 6.5 m/s. Total RNA extraction and purification were performed with TRIzol Plus RNA Purification Kit (ThermoFisher, USA), following the manufacturer’s instructions. RNA concentration was quantified with Qubit 2.0 Fluorometer and Qubit RNA Assay Kit, Broad Range (Invitrogen, USA), following the manufacturer’s instructions. RNA quality was determined by micro-fluidic capillary electrophoresis in the Agilent 2100 Bioanalyzer (Agilent Technologies, USA) using the RNA Nano6000 kit, and all RNA samples showed a RNA Integrity Number (RIN) ≥ 7.9 [36]. A pool of mRNA from livers of 6 control mice was prepared as described above, and served as a reference sample.

Total RNA (1 μg) was reversed transcribed using the iScript cDNA Syntesis Kit (Bio-Rad, USA), according to the manufacturer’s instructions.

### Quantitative real-time polymerase chain reaction (qPCR)

Genes and primers are listed in Table 1. Primers were designed using Primer-Blast (https://www.ncbi.nlm.nih.gov/tools/primer-blast/) or obtained from PrimerBank (https://pga.mgh.harvard.edu/primerbank/) [37]. Primer specificity was confirmed by analyzing melting curves from amplicons, and by performing agarose gel electrophoresis to discriminate PCR products. Amplified PCR fragments were visualized by ethidium bromide staining following electrophoresis in 1.5% agarose gels.

**Table 1:** Genes and primers used for gene expression analysis by qPCR.

Gene (NCBI access)	*Forward primer* (5’→3’) *Reverse primer* (5’→ 3’)	Amplicon length (bp)
*Rplp0* (Ribosomal protein, large, P0) (NM_007475.5)	AGATTCGGGATATGCTGTTGGC TCGGGTCCTAGACCAGTGTTC	109
*Vwf* (von Willebrand Factor) (NM_011708.4)	CTTCTGTACGCCTCAGCTATG GCCGTTGTAATTCCCACACAAG	125
*Adamts13* (ADAMTS13) (NM_001001322.2; NM_001290463.1)	AACACAGTGGTGGTGAAGCA ACAATTTCACCCCGAGGTCC	134
*P4hb* (Protein disulfide isomerase A1, PDI A1) (NM_011032.3)	GCCGCAAAACTGAAGGCAG GGTAGCCACGGACACCATAC	100
*F3* (Tissue factor) (NM_010171.3)	AACCCACCAACTATACCTACACT GTCTGTGAGGTCGCACTCG	101
*F7* (Factor VII) (NM_010172.4)	GCTGGACGCCAGATGGATAG AGTCATGTTCACCCATCACCA	92
*F8* (Factor VIII) (NM_007977.2; NM_001161373.1; NM_001161374.1)	AGAATCAAGCAAGCCGACCA ACGTGCTTTATACCTCTTGGCA	97
*F5* (Factor V) (NM_007976.3)	CCTGGTCAGCGCAACATCTA GCCTGCATCCCAGCTTGATA	105
*F10* (Factor X) (NM_001242368.1)	AGGCTGAGATAAGCAGAAGGC GGTTAATAAACACACCTTTCCCAGG	179
*F2* (Factor II) (NM_010168.3)	CCGAAAGGGCAACCTAGAGC GGCCCAGAACACGTCTGTG	103
*Thpo* (Thrombopoietin) (NM_009379.3; NM_001289894.1, NM_001173505.1)	CACAGCTGTCCCAAGCAGTA GGCTGTGACACTGAAGTTCG	97
*Tfpi* (Tissue factor pathway inhibitor) (NM_011576.1; NM_001177319.1; NM_001177320.1; NM_001355271.1; NM_001355273.1)	TGGAGCAGAAAGGCCAGATT TCAAAGTTGTTGCGGTTGCC	147
*Serpinc1* (Antithrombin III) (NM_080844.4)	GGCTGCTGGTGAGAGGAAG GGATTCACGGGGATGTCTCG	129
*Proc* (Protein C) (NM_008934.4; NM_001042767.3; NM_001042768.3; NM_001313938.1)	CCACCTGGGGAATATCTAGCA GAAGCTGTTGGCACGTCTG	101
*Plg* (Plasminogen) (NM_008877.3)	TGCAGTGGAGAAAAGTATGAGGG AGGGATGTATCCATGAGCATGT	102
*Serpinf2* (α_2_-antiplasmin) (NM_008878.2)	TTCTCCTCAACGCCATCCA GGTGAGGCTCGGGTCAAAC	62
*Fga* (Fibrinogen—α chain, variant 1) (NM_001111048.2)	GCCCAACGAGAGACTGTGAT TCTTGCCAGGTCCGGTTAAA	193
*Fgb* (Fibrinogen—β chain) (NM_181849.3)	ACGATGAACCGACGGATAGC CCGTAGGACACAACACTCCC	225
*Fgg* 1 (Fibrinogen—γ chain, variant 1) (NM_133862.2)	GACGGCATTATTTGGGCGAC AACGTCTCCAGCCTGTTTGG	141
*Fgg* 2 (Fibrinogen—γ chain, variant 2) (NM_001317105.1)	TCACCAAGGTGGTACTTACTCAAA TGATCCACGCTGACCTGTTT	192
*Hp* (Haptoglobin) (NM_017370.2; NM_001329965.1)	GCTATGTGGAGCACTTGGTTC CACCCATTGCTTCTCGTCGTT	101
*Mug1* (Murinoglobulin 1) (NM_008645.3)	GGCAGAGTTCTCCATAGATACCA TGCTTTGCACTTGCATGTCTT	126
*Nfkb1* (Nuclear factor of kappa light polypeptide gene enhancer in B cells 1) (NM_008689.2)	ATGGCAGACGATGATCCCTAC TGTTGACAGTGGTATTTCTGGTG	111
*Stat3* (Signal transducer and activator of transcription 3) (NM_213659.3; NM_213660.3)	CAATACCATTGACCTGCCGAT GAGCGACTCAAACTGCCCT	109

For qPCR reactions, cDNA samples were diluted to obtain 1–100 ng template per reaction, depending on the analyzed gene: 100 ng template per reaction for the genes *Vwf*, *Adamts13*, *F3*, *F7*, *F8*, *F5*, *F10*, *Tfpi*, and *Thpo*; 10 ng template per reaction for the genes *Rplp0*, *P4hb*, *F2*, *Serpinc1*, *Proc*, *Plg*, *Serpinf2*, *Fga*, *Fgg* 2, *Mug1*, *Nfkb1* and *Stat3*; 1 ng template per reaction for the genes *Fgb*, *Fgg* 1 and *Hp*. The reaction mixture contained cDNA templates (in 1–2 μL), 5 μL of 2X Fast SYBR Green Master Mix (ThermoFisher, USA), and 1 μL of gene-specific primers (0.2 μM final concentration; Invitrogen or Sigma, USA), in a final volume of 10 μL. PCR reactions were carried out in triplicate in 96-well format plates (ThermoFisher, USA) using a StepOnePlus Real-Time PCR System (Applied Biosystems, USA), in a total of 40 cycles, following manufacture instructions.

For the normalization of data, amplification efficiencies of six reference genes were tested (*Gapdh*, *Actb*, *Hprt*, *Rps29*, *B2m* and *Rplp0)*, and analyzed with geNorm [38]. *Rplp0* showed the best stability expression (M value = 0.281), and was chosen as the reference gene for the normalization of qPCR data. The relative gene expression in mice from saline control and BjV groups was analyzed by the 2^-ΔΔCT^ method, using the pool of mRNA from six normal mice, loaded in each experiment, as the reference sample [39]. Results are shown as relative mRNA levels based on 2^-ΔΔCT^ values. For statistical analyses, ΔΔCT values were used to compare experimental groups using ANOVA and correlation tests (see underneath).

### Statistical analyses

Sample size, i.e., the number of mice allocated in each experimental group, was based on previous data of experimental envenomation that resulted in falls in platelet counts and fibrinogen levels around 50–75% at 3 h after injection [33]. Normal distribution and homoscedasticity of results were analyzed using the software STATA^TM^, version 10, and data were transformed whenever necessary. For statistical comparisons among groups, two-way ANOVA was used, followed by Bonferroni *post-hoc* test. Results were considered statistically significant when p< 0.05. Data were expressed as mean ± standard error of mean (S.E.M.).

Scatterplots and Pearson’s correlation matrices were created to examine the correlation coefficients and linear regression between variables, taking into account data from control and experimental groups together; whenever statistically significant correlation coefficients were observed (p< 0.05 and correlation coefficients> 0.700), the data were split into control and experimental groups and re-analyzed independently to check if the significance was due to false correlations between both groups. Confidence intervals (95%) for correlation values were estimated using a percentile bootstrap, using 1000 replicates, and reported inside square brackets. Correlation coefficients, linear regressions and ANOVA analyses were performed in SPSS (version 22).

## Results

### Envenomation induces hemostatic disturbances

**Fig 1:**
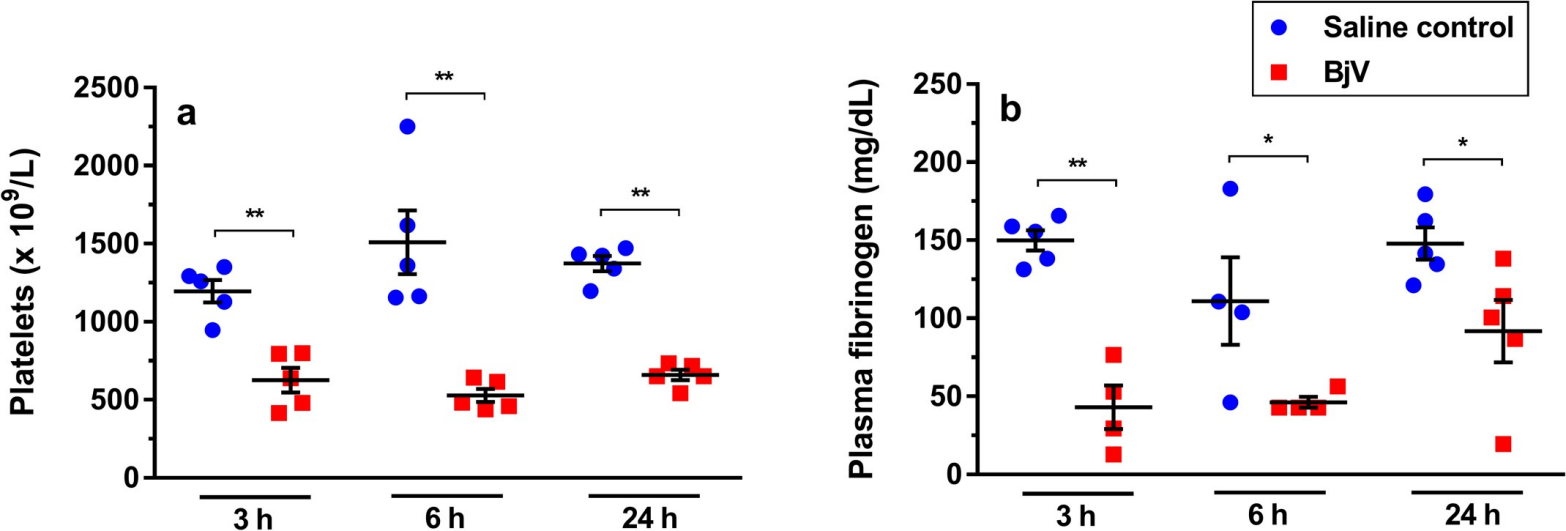
Platelet counts (**a**) and plasma fibrinogen levels (**b**) at 3, 6 and 24 h after injection of saline (saline control) or *Bothrops jararaca* venom (BjV). Two-way ANOVA, followed by Bonferroni *post-hoc* test were used to analyze data; * p< 0.05 and ** p< 0.001. Data were expressed as mean ± S.E.M. (n = 4-5/group).

As expected, platelet counts (Fig 1A) and fibrinogen levels (Fig 1B) steadily decreased after BjV injection. Platelet counts decreased around 50% at 3 and 24 h (p< 0.001), and showed a more evident decrease, around 65%, at 6 h (p< 0.001) in the BjV group. The drop (around 70%) in plasma fibrinogen levels was more evident at 3 h (p< 0.001). Although there was a tendency for recovery of fibrinogen levels over time, envenomed animals still showed hypofibrinogenemia at 6 and 24 h (p< 0.05).

### BjV induces expression of APP genes and the transcription factor *Stat3*

**Fig 2:**
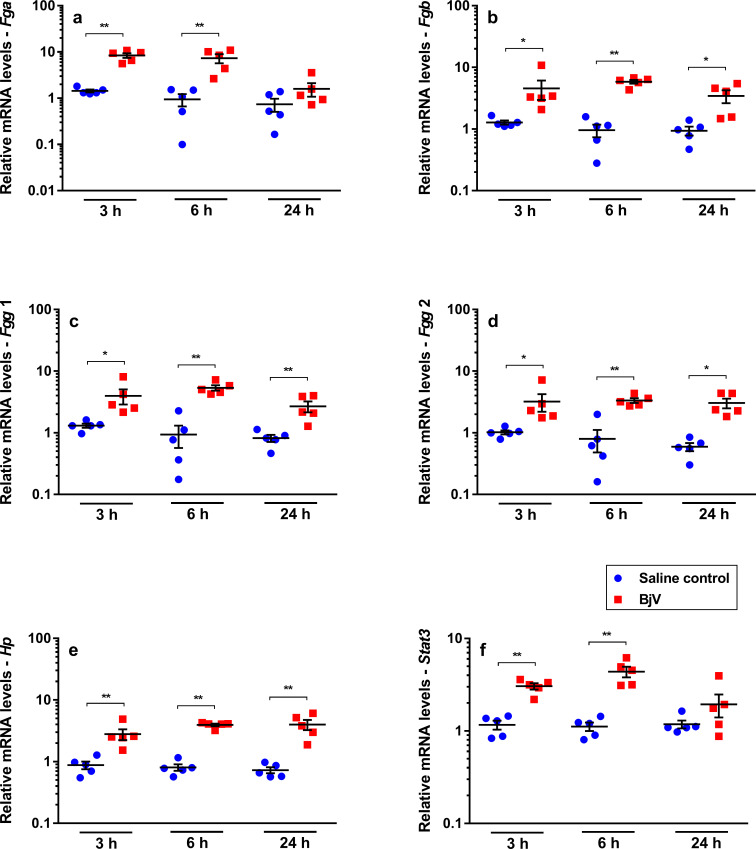
Relative mRNA levels of *Fga* (a), *Fgb* (b), *Fgg* 1 (c), *Fgg* 2 (d), *Hp* (e) and *Stat3* (f) genes highly altered in mouse livers at 3, 6 and 24 h after injection of saline (saline control) or *Bothrops jararaca* venom (BjV). mRNA levels were calculated using 2^-ΔΔCT^ method, using *Rplp0* as the reference gene. A pool of mRNA from six normal mouse livers was employed as the reference sample. Two-way ANOVA, followed by Bonferroni *post-hoc* test, were used to analyze data; * p< 0.05 and ** p< 0.001. Data were expressed as mean ± S.E.M. (n = 5/group, triplicates).

**Fig 3:**
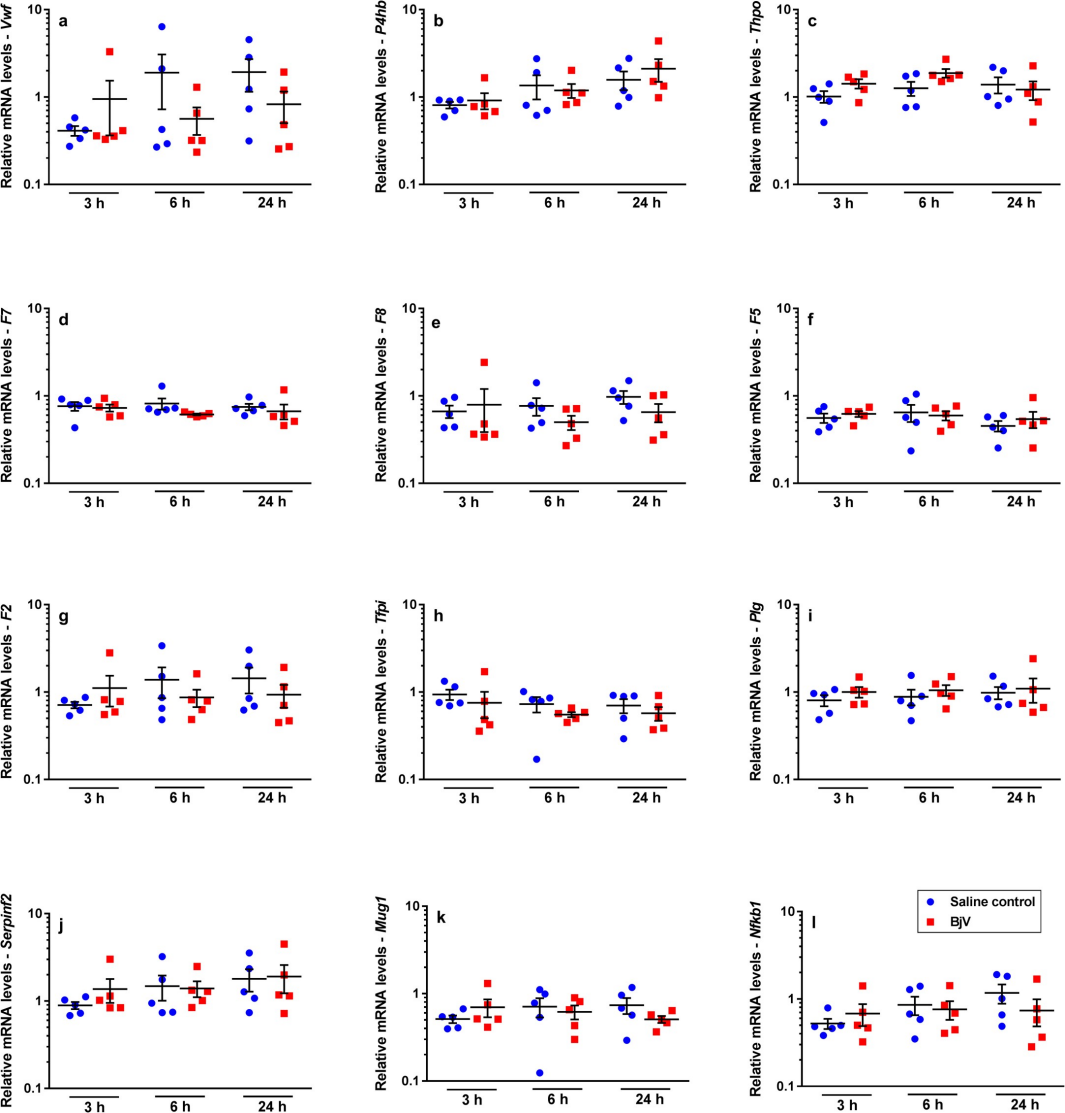
Relative mRNA levels of *Vwf* (a), *P4hb* (b), *Thpo* (c), *F7* (d), *F8* (e), *F5* (f), *F2* (g), *Tfpi* (h), *Plg* (i), *Serpinf2* (j), *Mug1* (k) and *Nfkb1* (l), genes that were marginally altered in mouse livers at 3, 6 and 24 h after injection of saline (saline control) or *Bothrops jararaca* venom (BjV). mRNA levels were calculated using 2^-ΔΔCT^ method, using *Rplp0* as the reference gene. A pool of mRNA from six normal mouse livers was employed as the reference sample. Two-way ANOVA, followed by Bonferroni *post-hoc* test, were used to analyze data; * p< 0.05 and ** p< 0.001. Data were expressed as mean ± S.E.M. (n = 5/group, triplicates).

Considering that BjV induces the synthesis of APP [18, 19], we studied fibrinogen gene expression [40], and observed that mRNA levels of fibrinogen chains were the most increased after venom injection. Expression of *Fga* (Fig 2A) only increased in the acute phase of envenomation (at 3 and 6 h, p< 0.001), whilst those of *Fgb* (Fig 2B), *Fgg* 1 (Fig 2C) and *Fgg* 2 (Fig 2D) were elevated at all time periods analyzed (p< 0.05), most evidently at 6 h (a 6-fold increase, p< 0.001). BjV also enhanced the expression of another APP gene, namely haptoglobin (*Hp*), which showed a 4 to 6-fold increase at all time intervals (p< 0.001, Fig 2E). On the other hand, the negative APP gene *Mug1* (Fig 3K), encoding murinoglobulin–which is a key serpin inhibitor in rodents [41] and that inhibits various venom activities *in vivo* [42]–showed no statistically significant differences between groups over time.

The cross-talk between the transcription factors STAT3 and NF-κB has an important role in the regulation of APP gene expression [43]. Interestingly, *Stat3* mRNA levels (Fig 2F) were increased at 3 and 6 h (p< 0.001) after envenomation, whilst *Nfkb1* mRNA levels (Fig 3L) showed no statistically significant differences between groups over time.

STAT3 and thrombopoietin have also been involved in the regulation of platelet signaling, activation and production [44, 45]. In addition, the JAK2-STAT3 pathway was shown to be involved in the increased hepatic *Thpo* mRNA levels and control of thrombopoiesis [45]. Even though thrombocytopenia occurred rapidly after envenomation, no change in *Thpo* mRNA levels (Fig 3C) was noticed over time. Besides, no statistically significant correlation (p> 0.05) was observed between gene expression of *Thpo*, *Stat3* and platelet counts.

### Gene expression of fibrinogen chain genes are correlated

**Fig 4:**
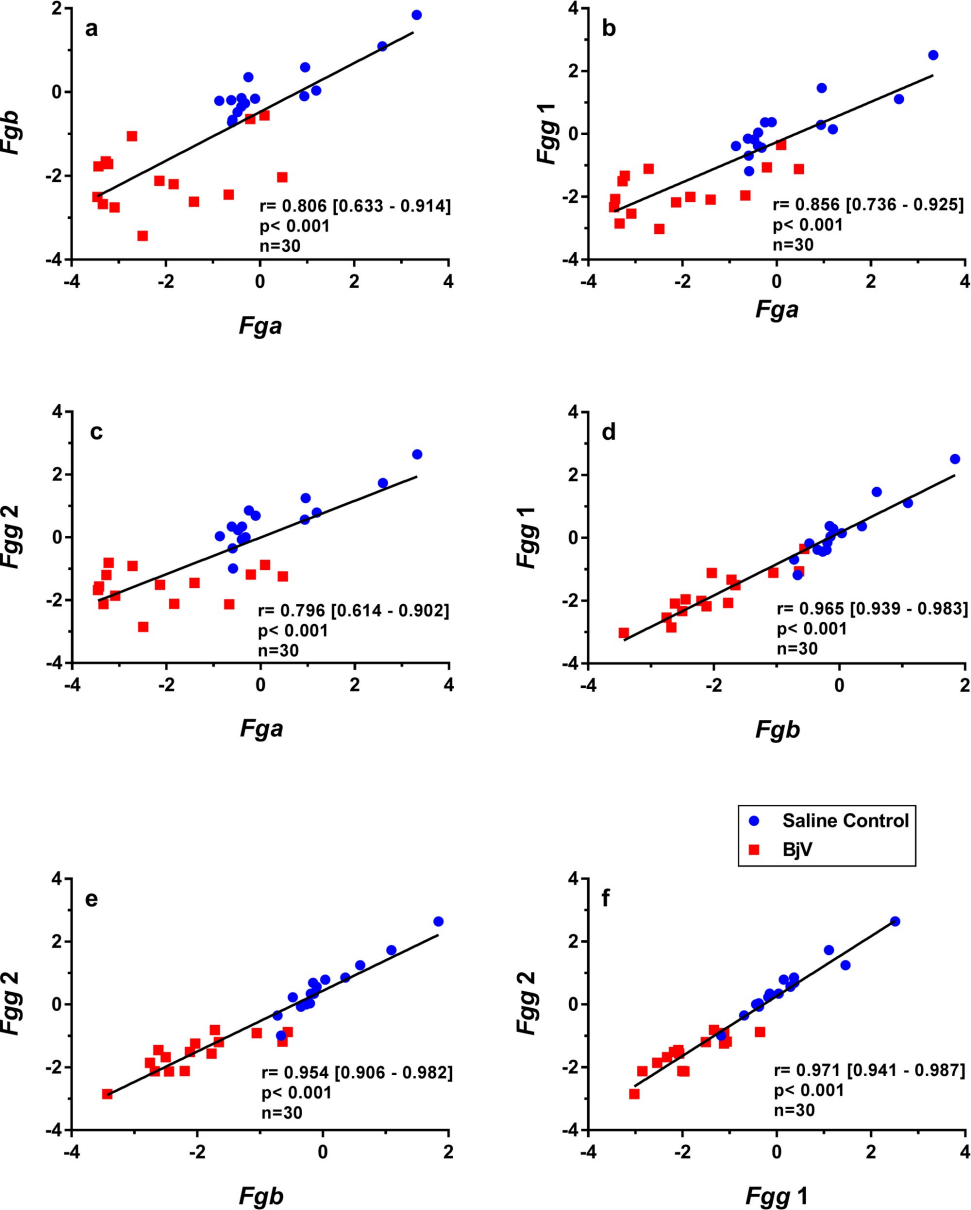
Scatterplots and linear regressions of paired observations between relative mRNA levels of the fibrinogen chain genes *Fgb* and *Fga* (a), *Fgg* 1 and *Fga* (b), *Fgg* 2 and *Fga* (c), *Fgg* 1 and *Fgb* (d), *Fgg* 2 and *Fgb* (e), *Fgg* 2 and *Fgg* 1 (f) in mouse livers at 3, 6 and 24 h after injection of saline (saline control) or *Bothrops jararaca* venom (BjV). mRNA levels were calculated using 2^-ΔΔCT^ method, using *Rplp0* as the reference gene. A pool of mRNA from six normal mouse livers was employed as the reference sample. Data were expressed as relative mRNA levels based on ΔΔCT values (n = 30). Pearson’s correlation was used, and results were expressed as correlation coefficient (r) and 95% confidence interval between square brackets.

Fibrinogen synthesis seemed a coordinated process, once mRNA levels of fibrinogen chains correlated significantly among each other (Fig 4A–4F) (r = 0.796–0.971, p< 0.001, n = 30). However, when correlation analyses were performed with split data between control and experimental groups, different results were obtained for *Fga*. At basal conditions of the control groups, the statistical significance of correlations was maintained between *Fga* and *Fgb* (r = 0.897 [95% confidence interval: 0.510–0.969], p< 0.001, n = 15), *Fgg1* (r = 0.859 [0.594–0.951], p< 0.001, n = 15) and *Fgg2* (r = 0.880 [0.564–0.963], p< 0.001, n = 15). In contrast, regarding the BjV group, *Fga* expression only predicted 3–30% of the variance of *Fgb* and *Fgg*, unveiled by weak correlation coefficients (r = 0.192–0.570, p = 0.026–0.493, n = 15) among these genes. These findings indicate that the regulatory mechanisms of *Fga* transcription may differ from those of other fibrinogen chains during envenomation.

### Hemostasis-related genes are differently expressed during envenomation

**Fig 5:**
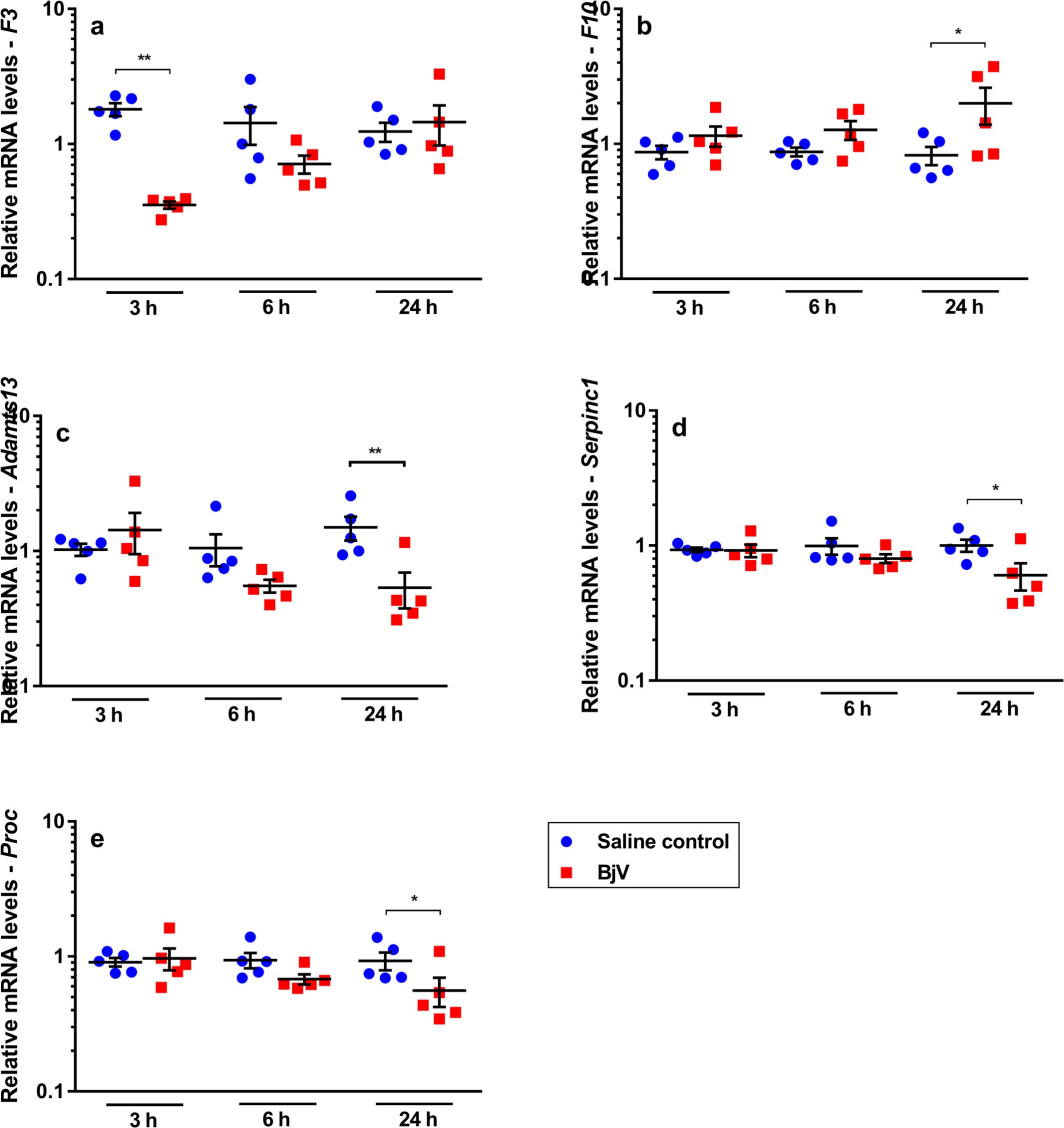
Relative mRNA levels of *F3* (a), *F10* (b), *Adamts13* (c), *Serpinc1* (d) and *Proc* (e), genes that showed alterations in mouse livers at 3, 6 and 24 h after injection of saline (saline control) or *Bothrops jararaca* venom (BjV). mRNA levels were calculated using 2^-ΔΔCT^ method, using *Rplp0* as the reference gene. A pool of mRNA from six normal mouse livers was employed as the reference sample. Two-way ANOVA, followed by Bonferroni *post-hoc* test, were used to analyze data; * p< 0.05 and ** p< 0.001. Data were expressed as mean ± S.E.M. (n = 5/group, triplicates).

Few authors have studied hepatic gene expression of hemostasis-related factors after acute experimental procedures or acquired conditions. In our model, using a snake venom that induces profound and rapid hemostatic changes in victims, hepatic expression of hemostasis-related genes (Figs 5 and 3) showed variable results. Interestingly, most of the studied genes did not display a statistically significant difference among groups over time (Fig 3).

*Bothrops* snake venoms induce protein expression and increased activity of tissue factor (TF) [25, 46, 47], but surprisingly a rapid and gross decrease in *F3* gene expression was noticed in the liver. In fact, *F3* (Fig 5A) was the only coagulation factor whose expression decreased significantly (p< 0.001) in the BjV group at 3 h, returning gradually to levels similar to controls only at 24 h.

In regard to other coagulation factors, envenomation also induces alterations in coagulation factors X, II, V and VIII, but, interestingly, only *F10* mRNA levels (p< 0.05, Fig 5B) increased at 24h after envenomation. On the other hand, gene expression of *F7* (Fig 3D), *F8* (Fig 3E), *F5* (Fig 3F) and *F2* (Fig 3G) did not change over time. Although plasma levels of antithrombin III remain constant during envenomation [20, 48], at 24 h an unexpected decrease in the mRNA levels of anticoagulant factors antithrombin III (*Serpinc1*, p< 0.05, Fig 5D) and protein C (*Proc*, p< 0.05, Fig 5E) was noticed, as well as a decrease in gene expression of the VWF-cleaving protease ADAMTS13 (*Adamts13*) (p< 0.001, Fig 5C).

**Fig 6:**
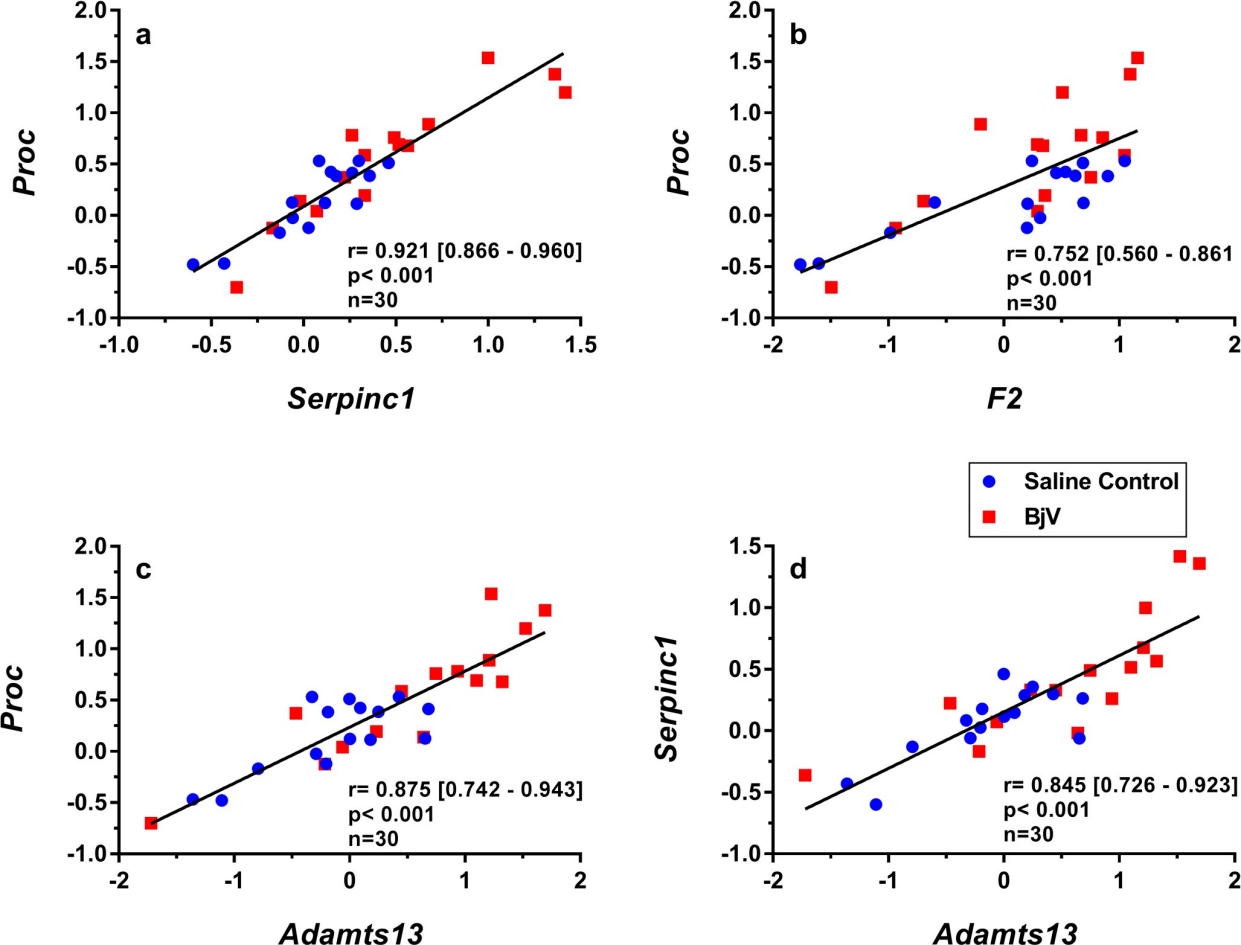
Scatterplots and linear regressions of paired observations between relative mRNA levels of hemostasis-related genes *Proc* and *Serpinc1* (a), *Proc* and *F2* (b), *Proc* and *Adamts13* (c) and *Serpinc1* and *Adamts13* (d) in mouse livers at 3, 6 and 24 h after injection of saline (saline control) or *Bothrops jararaca* venom (BjV). mRNA levels were calculated using 2^-ΔΔCT^ method, using *Rplp0* as the reference gene. A pool of mRNA from six normal mouse livers was employed as the reference sample. Data were expressed as relative mRNA levels based on ΔΔCT values (n = 30). Pearson’s correlation was used, and results were expressed as correlation coefficient (r) and 95% confidence interval between square brackets.

**Fig 7:**
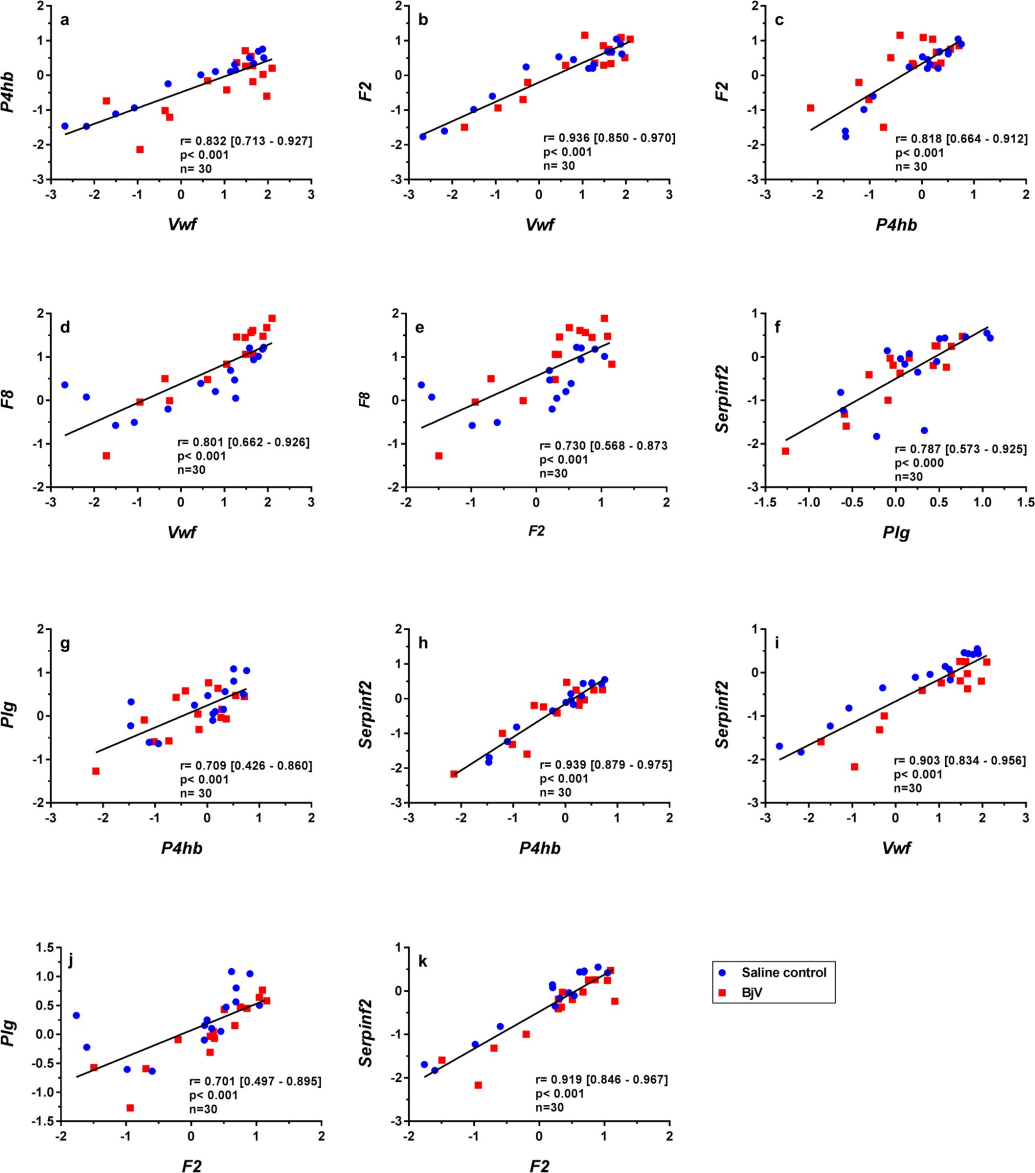
Scatterplots and linear regressions of paired observations between relative mRNA levels of hemostatic genes *P4hb* and *Vwf* (a), *F2* and *Vwf* (b), *F2* and *P4hb* (c), *Vwf* and *F8* (d), *F2* and *F8* (e), *Serpinf2* and *Plg* (f), *Plg* and *P4hb* (g), *Serpinf2* and *P4hb* (h), *Serpinf2* and *Vwf* (i), *Plg* and *F2* (j) and *Serpinf2* and *F2* (k) in mouse livers at 3, 6 and 24 h after injection of saline (saline control) or *Bothrops jararaca* venom (BjV). mRNA levels were calculated using 2^-ΔΔCT^ method, using *Rplp0* as the reference gene. A pool of mRNA from six normal mouse livers was employed as the reference sample. Data were expressed as relative mRNA levels based on ΔΔCT values (n = 30). Pearson’s correlation was used, and results were expressed as correlation coefficient (r) and 95% confidence interval between square brackets.

Although different patterns of gene expression were noticed over time, significant correlations were observed between relative mRNA levels (Figs 6 and 7). mRNA levels of the anticoagulants *Proc* and *Serpinc1* had strong correlations (Fig 6A). *Proc* expression also correlated with *F2* (Fig 6B) and *Adamts13* (Fig 6C), and *Serpinc1* correlated with *Adamts13* (Fig 6D).

The functional association between VWF and factor VIII is well-established at the protein level since VWF, the plasma carrier of factor VIII, is also involved in the regulation of FVIII activity [49]. In addition, PDIA1 (encoded by *P4hb*) was shown to be involved in the dimerization of VWF, a key step for VWF regulation [50]. Interestingly, these and other associations were also observed at a gene level herein. Thus, strong correlations between gene expression of *Vwf* and *P4hb* (Fig 7A), *Vwf* and *F2* (Fig 7B), *P4hb* and *F2* (Fig 7C), *Vwf* and *F8* (Fig 7D), and *F2* and *F8* (Fig 7E) were noticed.

Expression of two genes encoding major proteins of the fibrinolytic system–α_2_-antiplasmin (gene *Serpinf2*) and plasminogen (gene *Plg*)–was significantly correlated (Fig 7F), as well as with other genes: *Plg* and *P4hb* (Fig 7G), *Serpinf2* and *P4hb* (Fig 7H), *Serpinf2* and *Vwf* (Fig 7I), *Plg* and *F2* (Fig 7J), and *Serpinf2* and *F2* (Fig 7K).

Hemostasis-related factors in the liver show different responses to envenomation. However, regulation of some genes seems to be associated and share common pathways of regulation.

### Expression of transcription and hemostatic-related factors is correlated

To better understand the correlation in gene expression between the aforementioned genes, scatterplots from data of mRNA levels of hemostasis-related genes and the transcription factors *Nfkb1* and *Stat3* were also analyzed.

**Fig 8:**
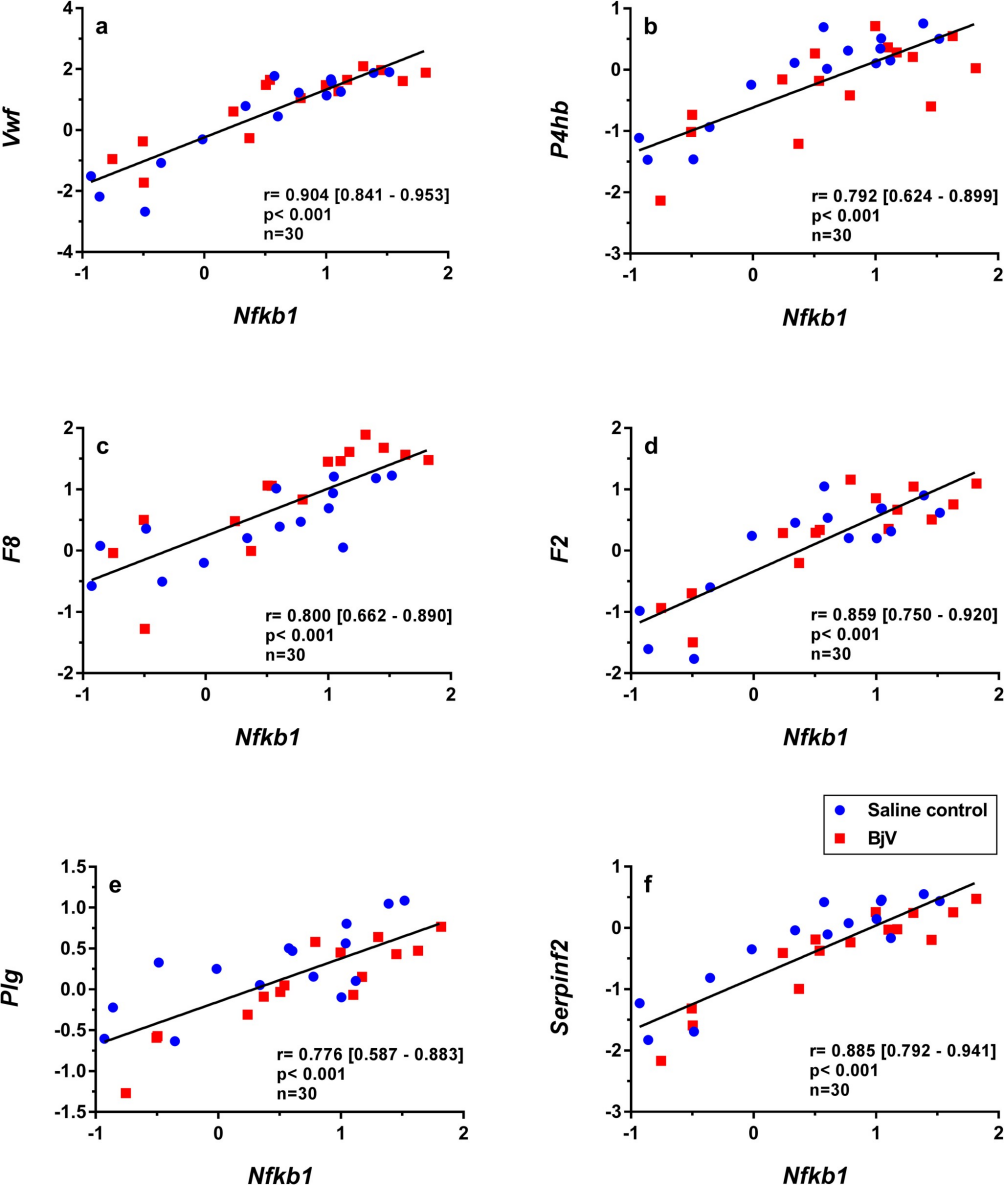
Scatterplots and linear regressions of paired observations between relative mRNA levels of hemostasis-related genes and *Nfkb1*, *Vwf* and *Nfkb1* (a), *P4hb* and *Nfkb1* (b), *F8* and *Nfkb1* (c), *F2* and *Nfkb1* (d), *Plg* and *Nfkb1* (e), *Serpinf2* and *Nfkb1* (f) in mouse livers at 3, 6 and 24 h after injection of saline (saline control) or *Bothrops jararaca* venom (BjV). mRNA levels were calculated using 2^-ΔΔCT^ method, using *Rplp0* as the reference gene. A pool of mRNA from six normal mouse livers was employed as the reference sample. Data were expressed as relative mRNA levels based on ΔΔCT values (n = 30). Pearson’s correlation was used, and results were expressed as correlation coefficient (r) and 95% confidence interval between square brackets.

NF-κB1 is a transcription factor that plays an important role in immunity and inflammation [43]. NF-κB1 may be activated by several stimuli, such as pro-inflammatory cytokines and oxidative stress and regulates genes related to hemostasis and inflammation [51]. Interestingly, important correlations were noted for *Nfkb1* mRNA levels and hemostasis-related genes, e.g., *Vwf* (Fig 8A), *P4hb* (Fig 8B), *F8* (Fig 8C) and *F2* (Fig 8D). Moreover, correlations were observed between gene expression of *Nfkb1* and the fibrinolytic proteins *Plg* (Fig 8E) and *Serpinf2* (Fig 8F). Using the coefficient of determination (r^2^) as index to predict this variation, these data suggest that *Nfkb1* accounted for 60–80% of the expression variance of important hemostatic genes, indicating that *Nfkb1* could be a relevant gene involved in the regulation of hemostasis-related genes.

## Discussion

Envenomation caused by *Bothrops jararaca* snakebites induces clinical alterations in bitten patients, as hematological and hemostatic disturbances [16, 20]. The recovery of these parameters over time is not completely elucidated, as well as the venom-induced hepatic response. Herein we present new insights into the complex regulation of gene expression of hemostasis-related factors.

In order to validate our experiment model, we first analyzed the occurrence of thrombocytopenia and hypofibrinogenemia, two major characteristic hemostatic disorders resulting from *B*. *jararaca* envenomation. Thrombocytopenia is an important hematological alteration that is related to the severity of envenomation, as well as to the development of systemic bleedings and edema. In agreement with previous results, thrombocytopenia and hypofibrinogenemia are not correlated, however the association of these disturbances increases the risk of bleedings during envenomation [46, 52, 53].

In models of acute immune thrombocytopenia [45] or non-immune thrombocytopenia [54], platelet counts are inversely related to plasma thrombopoietin levels, which are elevated independently of hepatic *Thpo* mRNA levels. Based on our results, the restoration of platelet counts during *B*. *jararaca* envenomation does not seem to be regulated by an increase in *Thpo* gene expression in the liver. In agreement with these results, rabbits had a minor increase in reticulated platelets in circulating blood following injection of BjV [48].

Hypofibrinogenemia is the result of the action of BjV toxins, such as SVSP and SVMP, that activate coagulation factors and lead to the formation of intravascular thrombin. The slow and constant activation of the coagulation cascade induces the consumption of coagulation factors, mainly fibrinogen, generating intravascular fibrin, which in turn, is degraded by the fibrinolytic system, generating a marked increase in fibrinogen/fibrin degradation products (FDP) [15, 21, 52, 55]. As expected in our model after 24 h of envenomation, the levels of fibrinogen tended to increase without the use of antivenom, which is also observed in patients bitten by *B*. *jararaca* snakes and treated with antivenom [55, 56]. The increment in the rate of fibrinogen synthesis after defibrinogenation induced by *B*. *jararaca* envenomation may be explained by the augmented synthesis of fibrinogen chains, as demonstrated by the elevated mRNA levels of fibrinogen chains in hepatocytes. Augmented and coordinated gene expression of fibrinogen chains was also observed in rat livers following infusion of the thrombin-like enzyme from *Calloselasma rhodostoma* snake venom [57]. The coordinated hepatic synthesis of three fibrinogen chains is well-described to be stimulated by the presence of FDP fragments in circulation [58–60], and due to the increase in circulating cytokines, mainly interleukin-6, during inflammation models [40, 61]. During *Bothrops* envenomation, cytokines and immune modulators are overexpressed [18], and FDP levels raise steadily, and in concert they might upregulate fibrinogen synthesis. That is, hepatocytes may increase synthesis rates when stimulated by components endogenously generated as a response to envenomation.

Furthermore, we showed that not only was that the gene expression of fibrinogen chains upregulated by envenomation, but also that of haptoglobin. Indeed, the stimulation of fibrinogen and haptoglobin genes is a well-known positive acute-phase response (APR) [62]. APR is an important systemic reaction evoked by diverse inflammatory conditions and tissue damage, like those occurring during envenomation [63, 64]. The increase in cytokine levels in such conditions may in turn lead to alterations in the transcription of APR genes in the liver. Our results are in agreement with the main APR features, i.e., the upregulation of positive APP genes, such as fibrinogen and haptoglobin, as well as the downregulation of negative APPs genes, e.g. protein C, antithrombin III, and murinoglobulin 1 [65–67].

During *B*. *jararaca* envenomation, the contribution of the inflammatory reaction or raised FDP levels to the upregulation of gene expression of fibrinogen chains and other APP in liver is difficult to separate. Infusion of the *C*. *rhodostoma* thrombin-like enzyme induced a rapid increase in *Fga*, *Fgb* and *Fgg*, peaking at 12–16 h [57]. On the other hand, asseptic inflammatory stimuli, such as burn injury [61] or turpentine injection [68], induced a peak in fibrinogen chains and *Hp* around 24 h post-stimuli. Our findings showed that the relative mRNA levels of *Fga*, *Fgb*, *Fgg1*, *Fgg2*, and *Hp* were already elevated at 3 h, and tended to peak at 6 h. Thus, both the inflammatory reaction and the rapid rise in FDP levels tended to accelerate the synthesis of these genes during *B*. *jararaca* envenomation. Moreover, secondary infections, which occur in some patients bitten by *B*. *jararaca* at later periods [69], might upregulate the mRNA synthesis of fibrinogen more strinkingly.

Reasoning about important transcription factors in hepatocytes, which could be linked to APR, we evaluated gene expression of STAT3 and NF-κB1. Hepatic STAT3 functions as a mechanism for the control of systemic inflammation, and upon stimuli IL-6 activates STAT3, which in turn induces the transcription of APPs genes [62, 70]. In fact, *Stat3* expression was indeed increased in the acute-phase of *B*. *jararaca* envenomation. IL-6 signaling not only activates STAT3, but also induces *Stat3* expression, via IL-6 responsive element in the gene promoter, explaining thereby the *Stat3* mRNA increase in hepatocytes during envenomation and the generation of an APR [71, 72].

Our results showed that neither *Fga* mRNA expression nor plasma fibrinogen levels were strongly associated with the gene expression levels of other fibrinogen chains after venom injection. In rats, gene expression of *Fga* differs from that of *Fgb* or *Fgg*, since the response of *Fga* to IL-6 stimuli is not mediated by STAT3 [70, 73]. Even though expression of fibrinogen chain genes was increased as soon as 3 h after venom injection, restoration of the circulating fibrinogen levels began only after 24 h in mice not treated with antivenom administration. It is important to emphasize that the circulating fibrinogen levels are regulated by its production and degradation, at both transcriptional and post-transcriptional levels [62, 70], but during envenomation venom enzymes that lead to fibrinogen consumption retard the restoration of fibrinogen levels in circulating blood.

Despite the coagulopathy observed following *Bothrops* envenomation, the hemostasis-related genes *Vwf*, *P4hb*, *Thpo*, *F7*, *F8*, *F5*, *F2*, *Tfpi*, *Plg*, *Serpinf2* and *Mug1* showed no quantitative difference in gene expression, but their expression was associated among each other and with *Nfkb1*. Interaction between transcription factors STAT3 and NF-κB is important for the transcriptional regulation of several genes related to modulation of inflammatory responses [43, 51]. However *Nfkb1* may also exert suppressor activity on inflammatory pathways [74] and our results suggest that *Nfkb1* may also be associated to the regulation of hemostasis-related genes. Moreover, our findings indicate that the restoration of prothrombin and factors V, VIII and X, which are decreased during envenomation [20, 21], seems to occur under basal hepatic conditions. In addition, it was demonstrated that hepatic expression of several genes may have common regulatory mechanisms and that the physiological synthesis rates of most factors remain unaltered even in the presence of anti-hemostatic and pro-inflammatory conditions, like snake envenomation-induced coagulopathy.

Circulating levels of factor VII were not altered during *B*. *jararaca* envenomation [25], and herein we showed that *F7* mRNA levels are neither altered. However, we showed for the first time a decrease in hepatic *F3* mRNA, which occurred as soon as 3 h after BjV administration, returning to control levels only at 24 h. TF expression is well known to differ depending on the tissue being analyzed, and to be regulated by several transcription factors, pathways and stimuli [75]. Interestingly, higher levels of constitutive TF expression seem to confer an additional hemostatic protection to specific tissues, such as lungs and heart. However, under physiological conditions, TF levels in the liver are considered comparatively low in regard to other organs, and in low-TF transgenic mice (which have only ≈ 1% of TF) the liver-specific hemostasis is considered normal, and those mice show no bleedings [75, 76]. Further studies are currently being carried out to understand the importance of TF alterations during envenomation.

Differently from the early pattern of *F3* gene expression during envenomation, gene expression of *Adamts13*, *Serpinc1* and *Proc* decreased, whilst that of *F10* increased at a later period of envenomation (24 h). Similar results were reported in an experimental model of direct liver hyperplasia and were associated to liver regeneration [77]. This may suggest that the impairment of anticoagulants during envenomation may be aggravated by the decrease in their hepatic synthesis. Interestingly, gene interactions as those of *Proc* and *Serpinc1*, and *Plg* and *Serpinf2* were already reported to be associated with liver function and liver regeneration [78, 79].

Although we could not evaluate all proteins whose genes have been studied due to limitations in the blood volume obtained from mice, it is important to emphasize that few studies have addressed how gene expression of various hemostatic and inflammatory factors synthetized by the liver occurs under steady state conditions and under acute systemic injuries. Understanding these mechanisms, using models that disturb both systems simultaneously, is important to increase our knowledge about how coagulation and inflammation are regulated under protein and gene levels.

In conclusion, our results demonstrate that envenomation by BjV induces different expression patterns of hemostasis-related genes. The association of gene expression of hemostasis-related factors, inflammatory proteins and transcription factors reaffirms the complex interactions that occur during envenomation. Moreover, the rapid induction of mRNA synthesis of fibrinogen chains explains the fast recovery of fibrinogen levels observed in bitten patients after antivenom therapy.

## Supporting information

S1 Table Raw data collected from control and BjV-injected mice. (PDF)
