# Supplementary material for: Liver gene regulation of hemostasis-related factors is altered by experimental snake envenomation in mice
Source: PLoS Negl Trop Dis. 2020 Jun 1;14(6):e0008379. doi: 10.1371/journal.pntd.0008379 (PMC7289449; doi:10.1371/journal.pntd.0008379)
Supplement: S1 Table — (PDF) [file pntd.0008379.s001.pdf]

3h = 0, 6h = 1, 24h=2
CT salina = 0, BjV=2

		Fibrinogêr	ΔΔCt vWF	2-ΔΔCt vWF	ΔΔCt ADAMTS13	2-ΔΔCt ADAMTS13	ΔΔCt F2	2-ΔΔCt F2	ΔΔCt F5	2-ΔΔCt F5	ΔΔCt F8	2-ΔΔCt F8
			DeltaVWF	2VWF	DeltaADAMS	2ADAMS	DeltaF2	2F2	DeltaF5	2F5	DeltaF8	2F8
CT salina	3h	158.87	1.574438731	0.33577373	0.002950033	0.997957282	0.68942	0.6201	1.18516	0.43978	1.20977	0.43234
CT salina	3h	138.22	1.139719009	0.453847964	-0.202441533	1.150643991	0.20005	0.87052	0.87543	0.54509	0.69201	0.61899
CT salina	3h	165.76	0.7883358	0.579011617	0.682755152	0.622974427	0.45545	0.72928	0.5782	0.6698	0.20369	0.86833
CT salina	3h	131.34	1.874797821	0.272665142	-0.187398911	1.138708843	0.90117	0.53545	1.36715	0.38766	1.18116	0.441
CT salina	3h	155.42	1.255247116	0.418921805	-0.290493011	1.223058161	0.31356	0.80466	0.40857	0.75337	0.05317	0.96382
BjV	3h	53.11	1.485602697	0.357099323	-0.060919444	1.043130346	0.29306	0.81617	0.42979	0.74237	1.06322	0.47856
BjV	3h	76.66	1.476343791	0.359398479	0.746888479	0.595887348	0.8562	0.5524	0.75609	0.5921	1.45145	0.36565
BjV	3h		1.60948054	0.327716328	-0.46570905	1.380995913	0.75361	0.59312	1.13791	0.45442	1.56608	0.33772
BjV	3h	12.88	-1.722325643	3.299678909	-1.721004804	3.296659317	-1.49405	2.81679	0.58949	0.66458	-1.27605	2.42176
BjV	3h	29.45	1.277783076	0.412428783	0.232647578	0.851071604	0.35597	0.78134	0.60142	0.6591	1.46085	0.36328
CT salina	6h	103.93	-2.678487778	6.401845136	-1.107067108	2.154072949	-1.76373	3.39575	2.09028	0.23483	0.35884	0.77979
CT salina	6h	46.21	-1.079527537	2.113343876	0.655464172	0.634871195	-0.59905	1.51472	-0.0683	1.04848	-0.50344	1.41759
CT salina	6h	183.03	1.77684021	0.291821845	0.426873525	0.743872091	1.0471	0.48394	0.81833	0.5671	1.01659	0.49428
CT salina	6h		1.230607351	0.426138011	0.180263519	0.882541779	0.20544	0.86727	0.17927	0.88315	0.47058	0.72168
CT salina	6h	110.77	1.900511424	0.267848399	0.24910895	0.841415937	0.61911	0.65107	1.00256	0.49911	1.22485	0.42784
BjV	6h	56.33	1.648143133	0.319050537	0.935764313	0.522765445	0.66986	0.62857	1.09189	0.46915	1.61074	0.32743
BjV	6h		0.609329224	0.655501404	1.102194468	0.465807422	0.28934	0.81827	0.46015	0.72691	0.48232	0.71583
BjV	6h	42.85	1.653260549	0.31792083	1.322875977	0.399737278	0.33779	0.79125	0.38711	0.76466	1.05736	0.48051
BjV	6h	42.85	-0.370986303	1.293236654	0.64202563	0.640812578	-0.69543	1.61937	0.67916	0.62453	0.50203	0.70611
BjV	6h	42.85	2.093559265	0.234301929	0.449860255	0.73211376	1.04578	0.48438	1.33991	0.39505	1.89214	0.26941
CT salina	24h	141.65	-2.182529449	4.539487564	-1.358145396	2.563554192	-1.60405	3.03995	1.9797	0.25354	0.0768	0.94816
CT salina	24h	121.05	0.453020096	0.730512014	0.092702866	0.937764216	0.53502	0.69015	0.80006	0.57433	0.39128	0.76245
CT salina	24h	134.78	1.668464661	0.314587955	-0.001897176	1.001315887	0.6846	0.62218	0.74263	0.59765	0.93673	0.52241
CT salina	24h	162.31	-0.301364899	1.23230972	-0.324878057	1.252558562	0.24226	0.84542	1.3188	0.40087	-0.19747	1.14668
CT salina	24h	179.57	-1.507750829	2.843663644	-0.791685104	1.731095245	-0.98291	1.97644	1.18529	0.43974	-0.57584	1.49055
BjV	24h	114.20	-0.949698766	1.931469326	-0.213498433	1.159496475	-0.93573	1.91285	0.0619	0.958	-0.04	1.02811
BjV	24h	100.51	1.971457163	0.254995349	1.524743398	0.347541364	0.50885	0.70278	0.96063	0.51383	1.67838	0.31243
BjV	24h	19.48	1.885865529	0.27058138	1.69377327	0.309117392	1.09462	0.46826	1.10369	0.46533	1.47686	0.35927
BjV	24h	86.87	-0.264530182	1.201244797	1.209852537	0.432312802	-0.20151	1.1499	1.98075	0.25336	-0.00559	1.00388
BjV	24h	138.22	1.049846649	0.483019504	1.228393555	0.426792416	1.15791	0.44816	0.94326	0.52006	0.83641	0.56004

		ΔΔCt F10	2-ΔΔCt F1	ΔΔCt Fib. A	2-ΔΔCt Fib	ΔΔCt Fib. B	2-ΔΔCt Fib	ΔΔCt Fib. (	2-ΔΔCt Fib	ΔΔCt Fib. (	2-ΔΔCt Fib. G2	ΔΔCt PLG	2-ΔΔCt PL
		DeltaF10	2F10	DeltaFGA	2FGA	DeltaFGB	2FGB	DeltaFGG:	2FGG1	DeltaFGG:	2FGG2	DeltaPLG	2PLG
CT salina	3h	0.53641828	0.68948	-0.317421595	1.2461	-0.26504612	1.20167	-0.43965	1.35627	0.00524	0.99637617	0.80404	0.57274
CT salina	3h	-0.16343753	1.11995	-0.862800598	1.81857	-0.20592499	1.15343	-0.38113	1.30236	0.03346	0.97707505	-0.09716	1.06967
CT salina	3h	0.13697688	0.90942	-0.395751953	1.31563	-0.34993617	1.2745	-0.37451	1.2964	-0.07179	1.05101764	0.05385	0.96336
CT salina	3h	0.7511495	0.59413	-0.394241333	1.31425	-0.14138667	1.10296	0.044	0.96996	0.34501	0.78730176	1.04947	0.48314
CT salina	3h	-0.04571851	1.0322	-0.594294866	1.50973	-0.7231458	1.65078	-0.68951	1.61274	-0.35291	1.27713553	0.10452	0.93011
BjV	3h	-0.05548795	1.03921	-3.427867889	10.762	-1.77298101	3.41759	-2.06903	4.19604	-1.56431	2.95735194	-0.03125	1.0219
BjV	3h	0.51956304	0.69758	-2.719961802	6.58855	-1.0513916	2.07253	-1.11176	2.16109	-0.90935	1.87819837	0.45209	0.73098
BjV	3h	0.10156059	0.93202	-3.22290802	9.33667	-1.71998088	3.29432	-1.33025	2.51445	-0.80726	1.74988279	0.47343	0.72025
BjV	3h	-0.89918264	1.86501	-2.49017779	5.61847	-3.43281873	10.7989	-3.0215	8.1201	-2.84804	7.200191	-0.57066	1.4852
BjV	3h	-0.28833262	1.22123	-3.272875468	9.66571	-1.65436681	3.14785	-1.50553	2.83929	-1.19939	2.29643327	-0.06647	1.04715
CT salina	6h	-0.05733426	1.04054	3.326091766	0.09971	1.841065089	0.27912	2.51325	0.17516	2.64062	0.16035918	0.32999	0.79554
CT salina	6h	0.00381279	0.99736	0.957581838	0.51492	0.594719569	0.66217	1.46125	0.36318	1.25064	0.42026074	-0.63375	1.55159
CT salina	6h	0.22241974	0.85713	-0.582584381	1.49753	-0.6643219	1.58482	-1.18113	2.26754	-0.99001	1.98619737	0.50392	0.70519
CT salina	6h	0.38791402	0.76423	-0.61054039	1.52683	-0.19206047	1.14239	-0.15012	1.10966	0.3456	0.78697846	0.15615	0.89742
CT salina	6h	0.50915337	0.70263	-0.103122711	1.0741	-0.15665563	1.1147	0.37445	0.7714	0.69162	0.61915635	1.0873	0.47064
BjV	6h	-0.24842262	1.18791	-3.088578542	8.50658	-2.75236384	6.7382	-2.53664	5.80234	-1.85438	3.61596234	0.15499	0.89814
BjV	6h	0.06632233	0.95507	-2.134955088	4.39223	-2.11658414	4.33666	-2.17844	4.52665	-1.50804	2.844227	-0.30819	1.23816
BjV	6h	-0.73726781	1.66702	-3.337306341	10.1072	-2.67357763	6.38009	-2.85224	7.22119	-2.12374	4.3582293	0.04927	0.96643
BjV	6h	-0.85212835	1.80516	-1.401655197	2.64205	-2.61671257	6.13351	-2.08739	4.2498	-1.44712	2.72664047	-0.59044	1.5057
BjV	6h	0.42496681	0.74486	-3.453258832	10.953	-2.50129	5.66191	-2.32855	5.02301	-1.67718	3.1980166	0.64036	0.64155
CT salina	24h	-0.0607001	1.04297	2.598885854	0.16507	1.090896606	0.46947	1.11043	0.46316	1.73195	0.30104396	-0.22149	1.16594
CT salina	24h	0.65065575	0.63699	-0.245780309	1.18573	0.359429677	0.77947	0.3681	0.7748	0.85527	0.5527605	0.47101	0.72146
CT salina	24h	0.59260941	0.66314	-0.475919088	1.3908	-0.47754542	1.39237	-0.17864	1.13182	0.23275	0.85100972	0.56413	0.67636
CT salina	24h	0.83489927	0.56062	1.191215515	0.43793	0.037680308	0.97422	0.14773	0.90267	0.79005	0.5783241	0.25181	0.83984
CT salina	24h	-0.27230453	1.20774	0.940603892	0.52101	-0.09860675	1.07074	0.29031	0.81773	0.56018	0.67821872	-0.60386	1.51978
BjV	24h	-1.90200233	3.73732	0.474120458	0.71991	-2.03139051	4.08799	-1.11642	2.16808	-1.24519	2.37049925	-1.26919	2.41026
BjV	24h	-1.65947787	3.15902	-1.834171295	3.56567	-2.19815954	4.58894	-2.00141	4.0039	-2.11944	4.34525844	0.43098	0.74176
BjV	24h	0.24682808	0.84275	0.094305674	0.93672	-0.55913226	1.47338	-0.35128	1.27569	-0.87727	1.83689615	0.76686	0.58769
BjV	24h	-0.51769765	1.43167	-0.66427358	1.58477	-2.4477609	5.45569	-1.95665	3.8816	-2.12912	4.37450722	-0.08971	1.06415
BjV	24h	0.2963028	0.81434	-0.205179214	1.15283	-0.64274788	1.5613	-1.06012	2.0851	-1.18188	2.26872625	0.58167	0.66819

		ΔΔCt A2AI	2-ΔΔCt A2	ΔΔCt STAT	2-ΔΔCt STAT3	ΔΔCt AT3	2-ΔΔCt AT	ΔΔCt PDI	2-ΔΔCt PD	ΔΔCt PC	2-ΔΔCt PC	ΔΔCt TF	2-ΔΔCt TF
		DeltaA2AI	2A2AP	DeltaSTAT	2STAT3	DeltaAT3	2AT3	DeltaPDI	2PDI	DeltaPC	2PC	DeltaTF	2TF
CT salina	3h	0.46017	0.7269	0.19232	0.875195334	0.1159	0.9228	0.51057	0.70195	0.12124	0.91939	-1.18925	2.28033
CT salina	3h	0.14543	0.90411	-0.36058	1.28394463	0.0268	0.9816	0.10614	0.92907	-0.12207	1.08829	-0.79995	1.74104
CT salina	3h	-0.03911	1.02748	-0.53496	1.448899859	0.26299	0.83336	0.1124	0.92505	0.41369	0.7507	-1.11776	2.17009
CT salina	3h	0.55006	0.68299	0.26144	0.834256485	0.17729	0.88436	0.75654	0.59191	0.3829	0.76689	-0.22054	1.16517
CT salina	3h	-0.16559	1.12163	-0.45328	1.369147828	-0.05917	1.04186	0.15303	0.89936	-0.02273	1.01588	-0.74917	1.68082
BjV	3h	-0.18926	1.14018	-1.66034	3.160900321	0.07126	0.95181	0.26435	0.83258	0.04006	0.97261	1.42035	0.37362
BjV	3h	0.25926	0.83551	-1.13406	2.194760811	0.49064	0.71171	0.71113	0.61084	0.75928	0.59079	1.86428	0.27466
BjV	3h	0.25408	0.83852	-1.55338	2.935039043	0.22315	0.85669	0.54976	0.68313	0.37156	0.77294	1.55	0.34151
BjV	3h	-1.59301	3.01678	-1.84855	3.601385578	-0.3618	1.28503	-0.7357	1.6652	-0.7023	1.62709	1.37999	0.38422
BjV	3h	-0.02955	1.0207	-1.73703	3.333479275	0.33145	0.79474	0.36457	0.7767	0.19412	0.87411	1.3428	0.39425
CT salina	6h	-1.69233	3.23177	0.31397	0.804422656	-0.59835	1.51398	-1.46007	2.75121	-0.47804	1.39285	0.84802	0.55555
CT salina	6h	-0.81585	1.76034	-0.52251	1.436457172	-0.06227	1.04411	-0.93412	1.91072	0.12639	0.91612	0.34036	0.78984
CT salina	6h	0.42295	0.7459	-0.30064	1.231686979	0.29911	0.81276	0.69453	0.61791	0.53053	0.6923	-1.59166	3.01395
CT salina	6h	0.07996	0.94608	-0.26711	1.203395995	0.28844	0.81879	0.31205	0.8055	0.11476	0.92354	-0.84993	1.80241
CT salina	6h	0.43808	0.73812	0.15116	0.900524167	0.35706	0.78075	0.50583	0.70426	0.38595	0.76528	-0.002	1.00139
BjV	6h	-0.02116	1.01477	-1.65599	3.151393623	0.26124	0.83437	0.28305	0.82185	0.781	0.58196	1.01035	0.49642
BjV	6h	-0.40985	1.32855	-2.29888	4.920756949	0.51666	0.69899	-0.1578	1.11558	0.69167	0.61914	0.26261	0.83358
BjV	6h	-0.37068	1.29296	-2.18038	4.532716885	0.56507	0.67592	-0.18021	1.13305	0.67978	0.62426	0.95333	0.51644
BjV	6h	-1.31364	2.48569	-2.62462	6.167204231	-0.0185	1.01291	-1.01617	2.02255	0.13896	0.90817	0.63859	0.64234
BjV	6h	0.24624	0.84309	-1.63408	3.103900173	0.33112	0.79492	0.20813	0.86566	0.58731	0.66558	-0.09678	1.06938
CT salina	24h	-1.82879	3.55239	-0.11697	1.084455861	-0.43006	1.34729	-1.46833	2.76702	-0.46839	1.38356	-0.04908	1.03461
CT salina	24h	-0.10592	1.07618	-0.15573	1.113987059	0.14751	0.90281	0.01375	0.99051	0.42314	0.7458	0.13555	0.91032
CT salina	24h	0.43788	0.73822	-0.12655	1.091680009	0.46069	0.72664	0.34395	0.78788	0.51151	0.70149	-0.59086	1.50615
CT salina	24h	-0.34958	1.27419	0.03688	0.974759084	0.08386	0.94353	-0.24435	1.18456	0.53085	0.69215	0.25293	0.83919
CT salina	24h	-1.22985	2.34543	-0.71032	1.636165724	-0.1293	1.09376	-1.11159	2.16083	-0.16982	1.12491	-0.92419	1.89762
BjV	24h	-2.16918	4.49768	-1.98036	3.9459102	-0.16802	1.12351	-2.13505	4.39252	-0.12423	1.08993	-1.72163	3.29808
BjV	24h	-0.19431	1.14418	-0.81737	1.762185312	1.41766	0.37432	-0.59683	1.51239	1.19901	0.43557	0.04417	0.96985
BjV	24h	0.47387	0.72003	0.19092	0.876046581	1.36024	0.38952	0.02564	0.98239	1.37595	0.3853	-0.5404	1.45438
BjV	24h	-0.99621	1.99475	-0.94841	1.929749854	0.67702	0.62545	-1.20828	2.31062	0.88931	0.53987	0.6042	0.65783
BjV	24h	-0.23522	1.17709	-0.23539	1.177222099	0.99932	0.50024	-0.41917	1.33716	1.53513	0.34505	0.17298	0.88701

		ΔΔCt TPO	2-ΔΔCt TP	ΔΔCt F7	2-ΔΔCt F7	ΔΔCt TFPI	2-ΔΔCt TF	ΔΔCt HAP	2-ΔΔCt HA	ΔΔCt MUG	2-ΔΔCt MI	ΔΔCt Nfkb	2-ΔΔCt Nfkb1
		DeltaTPO	2TPO	DeltaF7	2F7	DeltaTFPI	2TFPI	DeltaHAP	2HAPTO	DeltaMUG	2MUG1	DeltaNfkb	2Nfkb1
CT salina	3h	-0.32046	1.24873	0.17142	0.88797	0.39359	0.76123	0.86775	0.548	0.89414	0.53807	1.04643	0.48416
CT salina	3h	-0.50334	1.41749	0.1216	0.91917	-0.19989	1.14861	0.51413	0.70022	1.3357	0.3962	1.00426	0.49852
CT salina	3h	0.1574	0.89664	0.3618	0.77819	0.41048	0.75237	-0.3503	1.27483	0.5806	0.66869	0.33995	0.79007
CT salina	3h	0.01009	0.99303	0.33562	0.79244	0.52319	0.69583	0.16411	0.89248	0.87643	0.54471	1.389	0.38183
CT salina	3h	0.96371	0.51274	1.21179	0.43173	-0.41486	1.33317	0.04009	0.97259	1.29662	0.40708	1.12136	0.45966
BjV	3h	-0.75661	1.68952	0.3339	0.79339	0.3505	0.78431	-1.29074	2.44654	0.42132	0.74674	0.5064	0.70398
BjV	3h	0.21271	0.86291	0.75449	0.59276	1.0414	0.48586	-0.61956	1.53641	0.96075	0.51379	0.9968	0.50111
BjV	3h	-0.5868	1.50191	0.79903	0.57473	1.24355	0.42233	-1.36913	2.58315	1.27572	0.41302	1.62857	0.32341
BjV	3h	-0.87896	1.83905	0.09204	0.93819	-0.77916	1.71613	-2.28557	4.87557	-0.38581	1.3066	-0.49573	1.41004
BjV	3h	-0.29117	1.22364	0.41553	0.74974	1.48387	0.35753	-1.33336	2.51988	0.9744	0.50895	1.1014	0.46606
CT salina	6h	-0.88958	1.85263	0.48823	0.7129	2.5575	0.16987	0.35938	0.7795	3.01435	0.12376	-0.4858	1.40036
CT salina	6h	-0.24741	1.18707	0.52305	0.6959	0.2221	0.85732	0.4496	0.73225	0.01215	0.99162	-0.35446	1.27851
CT salina	6h	-0.79576	1.73599	-0.37227	1.29439	-0.01719	1.01199	-0.21102	1.15751	-0.15162	1.11082	0.5756	0.67101
CT salina	6h	0.398	0.75891	0.6187	0.65126	0.34897	0.78515	0.32545	0.79805	0.3549	0.78192	0.77662	0.58373
CT salina	6h	0.36592	0.77598	0.46237	0.72579	0.28297	0.8219	0.81728	0.56751	0.89864	0.53639	1.51942	0.34883
BjV	6h	-0.58787	1.50302	0.67098	0.62808	0.81593	0.56804	-2.0931	4.26663	1.74037	0.29929	1.1728	0.44356
BjV	6h	-0.82748	1.77458	0.61193	0.65432	0.60459	0.65766	-1.68381	3.21275	0.15889	0.89572	0.23855	0.8476
BjV	6h	-0.83765	1.78714	0.6981	0.61638	1.0063	0.49782	-2.03013	4.08442	0.61278	0.65394	0.53835	0.68856
BjV	6h	-1.43841	2.71022	0.79674	0.57565	1.15383	0.44943	-2.03667	4.10296	0.29986	0.81233	-0.50398	1.41812
BjV	6h	-0.70514	1.6303	0.75357	0.59313	0.77059	0.58618	-2.01898	4.05297	1.21354	0.43121	1.30393	0.40502
CT salina	24h	-1.13021	2.1889	0.35735	0.7806	1.77455	0.29229	0.21539	0.86131	1.77551	0.29209	-0.85871	1.81341
CT salina	24h	-0.02107	1.01471	0.75051	0.59439	0.14531	0.90418	0.81783	0.56729	0.81392	0.56883	0.60364	0.65809
CT salina	24h	0.31566	0.80348	0.47846	0.71774	0.99246	0.50262	0.79336	0.577	0.54847	0.68375	1.0391	0.48663
CT salina	24h	0.07578	0.94883	0.56361	0.67661	0.12664	0.91596	0.6018	0.65893	-0.23329	1.17552	-0.01472	1.01025
CT salina	24h	-0.99823	1.99755	0.04276	0.97079	0.16067	0.89461	0.03784	0.97411	0.05755	0.96089	-0.92979	1.90499
BjV	24h	-1.18527	2.27407	-0.22853	1.17164	0.55651	0.67994	-2.60733	6.09377	0.64638	0.63888	-0.75428	1.68678
BjV	24h	-0.14319	1.10435	0.71692	0.6084	1.42882	0.37144	-2.3741	5.18413	1.4537	0.36508	1.45295	0.36527
BjV	24h	0.95159	0.51706	1.12495	0.45852	1.37114	0.38659	-0.90643	1.8744	1.00347	0.4988	1.81734	0.28374
BjV	24h	-0.3954	1.31531	0.96443	0.51248	0.13252	0.91224	-1.93481	3.82327	0.80367	0.57289	0.37139	0.77304
BjV	24h	0.18699	0.87844	0.78577	0.58004	0.98438	0.50544	-1.57943	2.98851	1.10633	0.46447	0.79163	0.57769
